# Dynamic Monitoring of Mechano-Sensing of Cells by Gold Nanoslit Surface Plasmon Resonance Sensor

**DOI:** 10.1371/journal.pone.0089522

**Published:** 2014-02-21

**Authors:** Shu-Han Wu, Kuang-Li Lee, Ruei-Hung Weng, Zhao-Xian Zheng, Arthur Chiou, Pei-Kuen Wei

**Affiliations:** 1 Institute of Biophotonics, National Yang-Ming University, Taipei, Taiwan; 2 Research Center for Applied Sciences, Academia Sinica, Taipei, Taiwan; 3 Department of Mechanical and Mechatronic Engineering, National Taiwan Ocean University, Keelung, Taiwan; 4 Biophotonics and Molecular Imaging Research Center (BMIRC), National Yang-Ming University, Taipei, Taiwan; University of Waterloo, Canada

## Abstract

We demonstrated a real-time monitoring of live cells upon laminar shear stress stimulation via surface plasmon resonance (SPR) in gold nanoslit array. A large-area gold nanostructure consisted of 500-nm-period nanoslits was fabricated on a plastic film using the thermal-annealed template-stripping method. The SPR in the gold nanoslit array provides high surface sensitivity to monitor cell adhesion changes near the sensor surface. The human non-small cell lung cancer (CL1-0), human lung fibroblast (MRC-5), and human dermal fibroblast (Hs68) were cultured on the gold nanoslits and their dynamic responses to laminar shear stress were measured under different stress magnitudes from 0 to 30 dyne/cm^2^. Cell adhesion was increased in CL1-0 under shear flow stimulation. No adhesion recovery was observed after stopping the flow. On the other hand, MRC-5 and Hs68 decreased adhesion and recovered from the shear stress. The degree of recovery was around 70% for MRC-5. This device provides dynamic study and early detection of cell adhesion changes under shear flow conditions.

## Introduction

Understanding mechanobiology is crucial to understanding the living creature. Mechanical force not only induces structure change but also changes the function of tissue [1]. In the living body, there are some connate forces, such as blood pressure, breathing, bone support, and muscle contraction which complete the basic functions of life. Cells in the tissue are the basic unit as the reactor to the mechanical force. The biochemical responses to the mechanical loads play fundamental roles in the regulation of cell function and have been thoroughly explored [2]–[5]. The functional expression of cells induced by the mechanical stimulation is regulated by the signaling cascades of gene expression and protein synthesis. It brings about cell grow, death, proliferation [2], differentiation [6] and tissue remodeling [4], which are important to tissue homeostasis. In contrast, abnormal mechanical stimulation alters the cellular function and the extracellular matrix (ECM) composition, leading to organ pathologies such as osteoarthritis, tendinopathy, and fibrosis in bone, vessels, heart, lung, and skin [5], [7].

In the living body, the laminar shear stress generated by blood flow has been studied for the indication of cell functions and related to some pathology [8], [9]. In general, the cell would elongate and align parallel to the direction of flow in company with the focal adhesion alignment when shear stress is applied [10]. To respond to the laminar shear stress, cells alter their morphology and their distribution of cytoskeletal components [11]. The stress fiber networks (on the site of facing blood flow) and focal adhesion sites (at the basal side) have been recognized as the mechano-signaling complex that transmits the mechanosignal from the cell surface into the cell and activates the biochemical reaction given by mechanotransduction [12]. In order to understand the mechanism of mechanotransduction, numerous publications have focused on proteins expression, such as the expression of integrins [13], [14], G proteins [15], receptor tyrosine (RTKs) [16], cytoskeletons [17], stretch-activated ion channels [18], [19], mitogen-activated protein kinase (MAPKs) [20], and matrix metalloproteinase [8]. Following these studies, the cell focal adhesion distribution has been recognized as the initiator of mechano-induced signaling due to the adhesion protein expression regulated by mechanical force [12].

However, the impact of dynamic cellular response to mechanical stimulation is not fully understood [21]. In order to elucidate dynamic cellular response, new experimental techniques in cellular and sub-cellular detection are essential. These detection techniques can help identify the force sensors/receptors of cells for making the activation signal in cellular events [1]. There are many methods in the detection of gene expression, such as electrophoresis and the ELISA test for the quantitative analysis of protein amount [22], and flow cytometry in the detection of molecules on the cell membrane [19]. Lu *et al.* pioneered the study of surface coating on the substrate to cell adhesion by counting the cell number in video image under high fluidic shear force [23]. Nevertheless, this approach cannot control the variation in adhesion force between cells and it is hard to provide the information before cell detachment. Recently, Mott *et al.* monitored the cell cytoskeleton and focal adhesion complex regulated by unidirectional shear stress [8]. Tymchenko *et al.* used the composition of ridges and micropillar arrays as the force sensor to study the cell attachment by atomic force microscopy [24]. Hecht *et al.* used the atomic-fluorescence microscopy combined with a polydimethylsiloxane (PDMS) stretching system to perform the mechanotransduction study in the living cell [25]. However, these techniques require laborious procedures and fluorescence tagging.

The dynamic SPR detection system provides a real-time, label-free and highly sensitive detection to the refractive index change coming from the molecular interaction on the sensor surface. With these advantages, the SPR system is widely used and popular in diverse applications. The most popular and commercially used product is a prism-based SPR system, which couples an incident optical beam into surface plasmon polariton (SPP) at gold/medium interface [26]. Alternative to the prism-based SPR detection system is the nanostructure-based SPR system, which has advantages of small sensing area [27], chip-based, simple optical detection system, and ease in combining with microfluidic systems. The SPR property of metallic nanostructures was first demonstrated by Ebbesen *et al.* [28]. In the nanostructure-based SPR sensor, there are SPR and localized SPP resonance (LSPR) modes [29], [30]. The SPR in the metallic nanostructure has a lateral resolution of only a few microns [27], [31], [32]. This unique feature enables us to detect mechano-induced response from a small area. Moreover, the real-time SPR detection system can provide early detection before morphological changes of cells. Such information is hard to be obtained by other detection techniques. By monitoring cell adhesion changes, it is possible to measure earlier events of disease progression [8]. In this study, we first apply a gold nanoslit array-based SPR microfluidic device to measure the dynamic response of a small number of cells upon mechanical stimulation in real time without the need of labeling. The early detection is done by directly observing cell adhesion changes before the detachment of cells from the substrate.

## Materials and Methods

### Chemicals

Phosphate buffered saline 10X (PBS 10X) was purchased from Sigma-Aldrich. Human non-small cell lung cancer cell line (NSCLC), CL1-0, was acquired from Prof. Pan-Chyr Yang [33]. Human lung fibroblast (MRC-5) and dermal fibroblast (Hs68) were purchased from ATCC and cultured by the protocol. CL1-0 and Hs68 were cultured in the medium of DMEM supplemented with 10% fetal bovine serum (FBS) and 1% penicillin/streptomycin/amphotericin (PSA) solution in a 37°C with 5% CO_2_ incubator. The medium of MEM supplemented with 3 mM L-glutamine, Earle’s balanced salt solution (EBSS) containing 1.5 g/L sodium bicarbonate, 0.1 mM non-essential amino acids, 1.0 mM sodium pyruvate, and 10% FBS was for MRC-5 cell culture. Distilled deionized water (resistance = 18 MΩ) from Milli-Q integral system was used to prepare all aqueous solutions.

### Fabrication of Gold Nanoslit Array and Microfluidic Device

The gold nanoslit array was made on a polycarbonate substrate by a thermal-annealing template-stripping method [34]. Briefly, the silicon wafer with nanoslit structure in the period of 500 nm and with a slit-width of ∼40 nm was prepared for achieving maximum SPR sensitivity [35]. Next, we coated gold film (50-nm-thick) on the silicon template using an e-beam evaporator. Then the silicon template with coated gold film was used as the mold for imprinting gold nanoslits on the polycarbonate film. The nanoimprint was done with a home-built chamber with heat and pressure control. The following conditions were used: temperature = 170°C, pressure = 1.961×10^6^ Pascal, duration = 30 minutes. After cooling, the polycarbonate film was stripped away from the silicon template and the gold nanoslit array pattern was transferred to the polycarbonate film. In the template-stripping method, the nanostructure is uniform in a large area and performs at a higher sensitivity compared to other fabrication techniques [34]. For detecting the mechanical force sensing of cells *in situ*, the nanoslit array was combined with a PDMS microfluidic chamber. The depth of the chamber was 90 µm using standard photolithographic process.

### Cell Culture on Gold Nanoslits

To help cells adhere to the gold nanoslit array, we injected 100% FBS into the microchannels and incubated it for 2 hours. Afterwards, 1X PBS solution was used as the wash buffer for flushing away the unbinding FBS in the chamber. Finally, cells (2.5×10^5^ cells/mL) were flowed into the PDMS-sensor device at 5 µL/min for 10 minutes and cultured overnight.

### Cell Focal Adhesion Image

The actin cytoskeleton and focal adhesion staining kit was purchased from Millipore. The vinculin labeling fluorescence was done in accordance with the staining protocol. The cell was fixed with 4% paraformaldehyde in 1X PBS for15–20 minutes at room temperature. Then, cells were permeabilized by Triton X-100 in 1X PBS for1–5 minutes. 1X PBS containing 0.05% Tween-20 as a wash buffer was performed. 1% BSA in 1X PBS is the blocking solution to block the surface from nonspecific binding.

### Monitoring of Cell Response to Shear stress

The cell culture medium was flowed at 0.5 µL/min to remove the suspension cell before stressing them. We applied different flowing rates to regulate the strength of shear stress in the microchannels by using the size of flow chamber presented in following equation [36]:
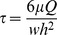
(1)Where τ is the strength of laminar shear stress in the unit of dyne/cm^2^ (1 Pascal = 10 dyne/cm^2^), and μ is the dynamic viscosity of solution. Here we use the cell culture medium DMEM, which is 0.0084 poise at 37°C [37], and *Q* is the velocity of flowing rate. *w* and *h* are the width and height of flow channel, respectively. The procedures are as following: first, a referenced signal is detected with *Q = *0 in order to obtain the initial state of cells for ∼5 minutes. After that, we applied shear stress to stimulate the cell by flowing culture medium at different flowing rate for 20 minutes. It is noted that the height of the channel was 90 µm which was much larger than the height of adhesion cells (3–5 µm). Since only a layer of cell was on the surface, the surface roughness was much smaller than the channel height. Therefore, the shear stress of the flow was simply estimated by equation (1). In the experiments, the τ values are 3, 7.5, 10, 15, and 30 dyne/cm^2^, respectively. Finally, we stopped the flowing and monitored the cell recovery for ∼ 40 minutes.

### Principle of Nanoslit SPR

In periodic gold nanostructures, the optical transmission spectrum is usually accompanied by an asymmetric resonance which is known as a Fano-like SPR profile. It is understood in terms of the coupling of broadband wave (a continuum state) with the surface-bound state of a periodic array (a discrete state) [30]. In the nanoslit array, the gap plasmon in the slit forms a broadband and localized SPP resonance [38]. The Bloch wave surface plasmon polariton (BW-SPP) in periodic nanostructures is a discrete resonance. Its resonant condition for one-dimensional periodic structures is described by [28].
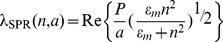
(2)where *P* is the period of the nanostructure, *a* is the resonant order, *ε_m_* is the dielectric constant of the metal, and *n* is the environmental refractive index. The position of Fano resonance can be predicted by equation (2). In our prior work, we have demonstrated that nanoslit array has a better sensitivity than commonly used nanohole arrays [29]. We also optimized the sensor sensitivity by considering the optical properties of gold and SPR conditions of the nanostructures. The optimal period of the nanostructure was 500 nm [35].

### Experimental Setup and Signal Analysis

**Figure 1:**
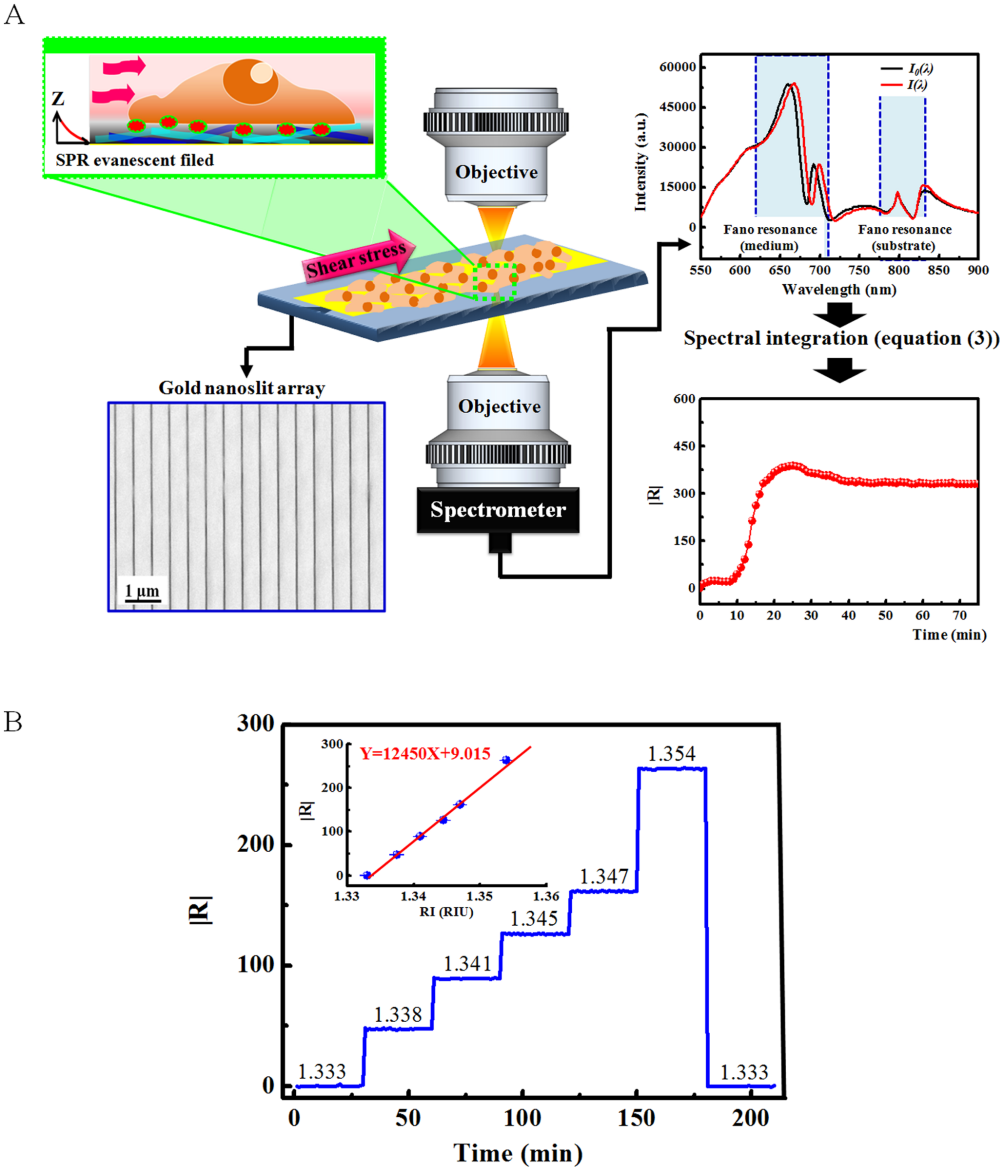
A schematic diagram of shear flow induced cell adhesion change in real-time and label-free measurement. (A) The target cells were cultured in a microfluidic-nanoslit SPR sensor device. The SEM image shows gold nanoslit structure on polycarbonate film. The cell adhesion condition under mechanical force stimulation was detected by Fano-like SPR in gold nanoslit array. The transmission spectra were detected by a spectrometer. The dynamic response curve is obtained by recording a sequence of spectra and calculated the response 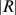 during the shear-flow interaction. *I_0_(λ)* is the referenced spectrum (before the mechanical stimulation) and *I(λ)* is the spectrum when cells were stimulated by the shear flow. (B) The response 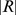 of the SPR sensor when different refractive index medium was applied to the SPR sensor. The 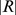 value is defined by the integration equation (equation (3)). FA: cell focal adhesion.

The cell adhesion measurement by nanoslit SPR sensor detection is illustrated in Figure 1A. The cell was cultured on the nanoslit array SPR sensor combined with the microfluidic device. The dynamic change of cell adhesion was monitored during the fluidic shear stress stimulation. The gold nanoslit array on the polycarbonate film fabricated by thermal-annealing template-stripping method was shown in the SEM image. The incident light was linearly polarized and focused on the sensor surface. We measured the transmission spectra as a function of time. In the figure, *I_0_(λ)* was the referenced transmission spectrum (initial state of cell) and *I(λ)* was the spectrum when cells were stimulated by the shear flow. Two Fano-like SPRs were measured, one occurred near 690 nm, the other was 800 nm. According to the geometry of the nanoslit SPR sensor, there are two interfaces for BW-SPP. For a 500-nm-period array, the resonant wavelength of the BW-SPP at the gold/medium interface is 704 nm (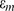 = −16+1.0*i* for gold at 700 nm, a = ±1, n = 1.3320 and P = 500 nm) and 799 nm at the substrate/gold interface (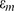 = −23+2.0*i* for gold at 800 nm, a = ±1, n = 1.520 and P = 500 nm) [39]. The measured two Fano-like SPRs were in good agreement with the predictions by equation (2). It is noted that the substrate/gold interface has no contact with the environment. The spectral shift causing by the SPR at this interface has no response to the external stimulation. On the other hand, the SPR has an obvious redshift at gold/medium interface. Therefore, we analyzed the response of the SPR near the gold/medium interface.

The analysis of the SPR resonant change was by spectral integration method [40] as indicated in equation (3).
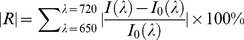
(3)Where R is the SPR repsonse, *I_0_(λ)* is the referenced spectrum and *I(λ)* is the measured spectrum. The sperctal integration region was selected from 650 nm to 720 nm, where the SPR occurred at the gold/medium interface. The advantage of spectral integration method is fully considering the signal of SPR resonant region (wavelength650–720 nm) both in wavelength shift and intensity change instead of single wavelength shift or intensity change at a particular wavelength. Intensities around the resonance peak could potentially all produce good intensity sensitivity. With the integration, the signal-to-noise (S/N) ratio is enhanced by smoothing noise from the light source and detector. From our previous results, the detection resolution is about 6 times higher than the commonly used wavelength or intensity methods [40]. It is noted that the quality factor of the SPR was not changed because most biomolecules have no absorption in the wavelength range of600–1000 nm. There is no additional surface plasmon propagation loss. The biomolecular layer on the sensor surface increases the surface refractive index. From equation (2), it results in the redshift of the SPR wavelength. Most SPR responses thus come from the wavelength shift.

The 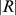 only shows the magnitude of SPR change. For the condition of surface index increase, such as the increase of cell adhesion, the SPR spectrum is red-shifted. On the contrary, if the cell adhesion is decreased, the spectrum is blue-shifted. Therefore, we also considered the direction of spectral shift in the signal analysis. We define the positive and negative R according to the spectral redshift and blueshift, respectively. The redshift indicates an increase of cell adhesion and the blueshift is a decrease of adhesion. Figure 1B shows the R value when different refractive index medium was applied to the SPR chip surface. The inset shows a linear dependence of R with the refractive index. The slope of sensing curve indicated that this sensor had a sensitivity of 12450 nm%/RIU (wavelength650–720 nm). Considering the noise floor, the sensor had a detection limit of about 2×10^−5^ RIU.

## Results and Discussion

### Correlation between Cell adhesion and SPR Response

**Figure 2:**
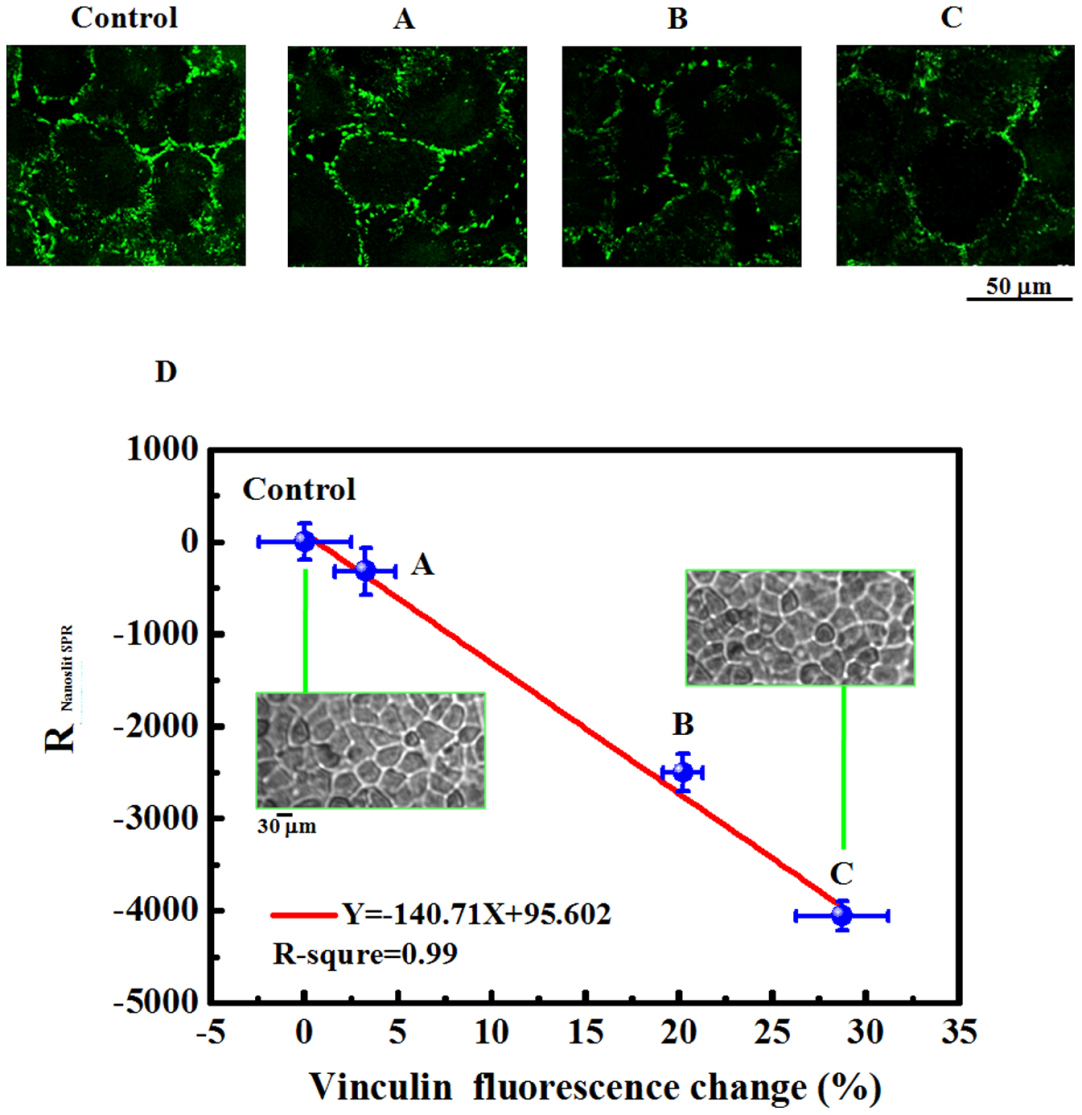
The correlation of nanoslit SPR response and cell focal adhesion presented by vinculin fluorescence signal. The vinculin fluorescence in green of CL1-0 was shown in confocal images (control, A–C). The fluorescence images, control, (A), (B), and (C) correspond to the data points, control, (A), (B), and (C) in (D), respectively. The inset in Figure 2D show the bright-field images of cells. The standard deviation was calculated from 3 repeated experiments.

The nanoslit SPR biosensor measured the surface refractive index change resulting from the cell adhesion change under fluidic-mechano stimulation. The cell attachment is the interaction of adhesion molecules between the cell membrane and the ECM and in the response to the shear stress activation [12]. In SPR detection, the detection field is a couple hundred nanometers away from the sensor surface, which is the main region of the interaction between ECM and receptors on cell membrane. The SPR signal comes from the change of surface refractive index (Δ*n*). A change of mass density of biomolecules, such as lipids and proteins, onto the chip surface changes Δ*n* and makes a spectral shift of the SPR. In the measurement of cell adhesion change under external stimulation, the Δ*n* is attributed to the change of cell focal adhesion. Therefore, we studied the relationship between the SPR signal and the focal adhesion proteins. The vinculin, one of the proteins in the cell focal adhesion complex, has been studied in relation to a mechanically induced response and cell invasion [41]. We use the fluorescence intensity of the vinculin to indicate the amount of focal adhesion. We applied the doxorubicin (DOX) in the culture medium to inhibit the focal adhesion formation in lung cancer cells (CL1-0) at different levels [42]. The vinculin signal decreased as the increase of DOX concentration. Figures 2A–C show the fluorescence images. The focal adhesion distribution was indicated in green. The cells at the same conditions were measured by nanoslit SPR and fluorescence labeling, respectively. The experiments were repeated 3 times. The mean values and the standard deviations are shown in the Figure 2D. The data points in Figure 2D were related to fluorescence images (control, A–C). The fluorescence intensity (V) in Figure 2 was normalized by the fluorescence intensity in the control (V_0_). The normalized difference between the fluorescence singals and the control is defined as ΔV/V_0_ (ΔV = V-V_0_). The signal that the nanoslit SPR detected was decreased with the decrease of ΔV/V_0_. The mean ΔV/V_0_ values, 3.24, 20.20, and 28.71 were corresponding to the SPR responses, −319.80, −2500.84, and −4056.25, respectively. The result shows the positive correlation between the SPR response and the amount of cell adhesion molecules (fluorescence vinculin signal). The fitting of the data points also indicates that 1% cell focal adhesion change induces 140.71% change of nanoslit SPR sensor response. It should be noted that the standard deviation in the nanoslit SPR detection is much smaller than the signal of vinculin fluorescence. It indicates that the nanoslit SPR detection has a better detection sensitivity. The inset in Figure 2D show the bright-field images of cells. The images indicate that the cell sizes did not change significantly while cell focal adhesion was changed about 30%. It is actually the advantage of the nanoslit SPR chip, providing detection of cell adhesion changes before the change in cell morphology.

### Dynamic Response of Cell Adhesion under Shear Stress Stimulation

**Figure 3:**
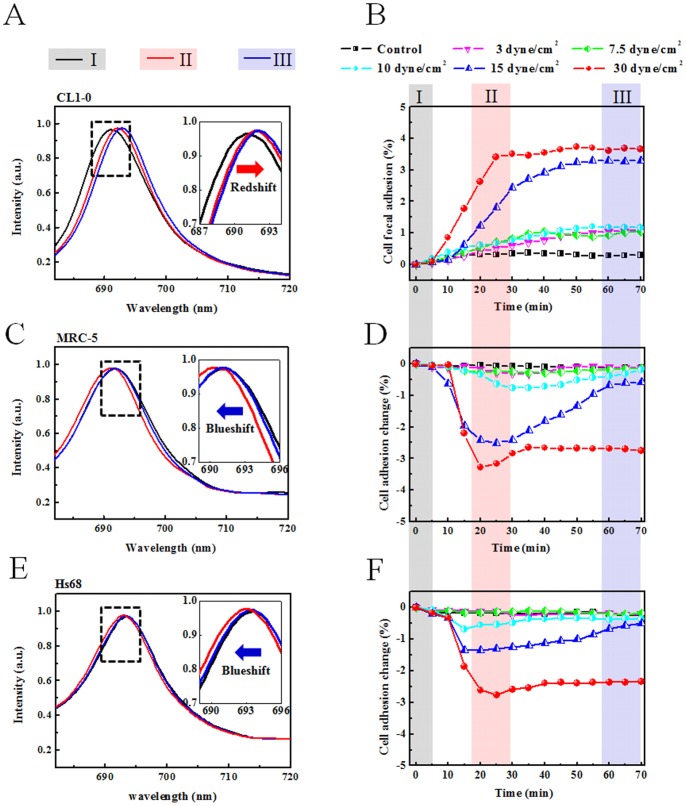
The dynamic curve of adhesion measurements for different kinds of cells: (A–B) CL1-0 (C–D) MRC-5 (E–F) Hs68. The shear stresses are 3, 7.5, 10, 15, and 30 dyne/cm^2^ applied in all three cell lines. The control experiment is the cell cultured in the microchannel without the medium flowing. (A, C, E) The spectra change under 15 dyne/cm^2^ shear stress stimulation. The black lines show the initial spectrum (I). The red lines show the maximum changes of spectrum (II). The blue lines show the final spectrum after stopping the flow (III). Note, redshift of spectrum in CL1-0 but blueshift in MRC-5 and Hs68. (B, D, F) The amounts of adhesion changes in dynamically for CL1-0, MRC-5, and Hs68 cells under shear stresses of 3, 7.5, 10, 15, and 30 dyne/cm^2^, respectively. The shear flow starts at 5 min and stops at about 25 min.

We performed the nanoslit SPR sensor in the cell response to shear stress stimulation using three different types of cells: human non-small cell lung cancer cell (CL1-0), human lung fibroblast (MRC-5), and human dermal fibroblast (Hs68). The size of SPR chip was 200×200 µm^2^. The cell number was about70–120 depended on the cell types for mechanical stimulation experiments. In Figure 3, the cells reacting to fluidic shear stress stimulation were recorded on a dynamic response curve. Figures 3A, 3C, and 3E show the representative spectra under shear stress of 15 dyne/cm^2^ for different interaction intervals: (I) the initial state, (II) the maximum response to shear stress, and (III) the stable state after stopping shear stress. The spectral shift at the SPR resonant wavelength is caused by the refractive index change, which is related to the amount of cell adhesion near the sensor surface. The redshift resulting from the refractive index increase indicates the increase of cell adhesion. The blueshift was due to the decrease of cell adhesion. CL1-0 cells increased adhesion with the increase of shear stress. On the contrary, the cell adhesion of MRC-5 and Hs68 (normal cell) was decreased in the sensor signal upon the shear stress stimulation. Figures 3B, 3D, and 3F show the dynamic adhesion changes of CL1-0, MRC-5, and Hs68 cells under shear stress of 3, 7.5, 10, 15, 30 dyne/cm^2^, respectively. The cell culture without flow, i.e. 0 dyne/cm^2^, was used as the control in all three cell lines. These adhesion changes were obtained by using the equation (3) and the fitting curve of Figure 2D. The maximum adhesion changes were under 5%. Cells do respond to the fluidic shear stress activation but in different levels. During the flow shear stress stimulation, the absolute adhesion changes increased gradually by the time and reached to the maximum value. The maximum changes in cell focal adhesion of CL1-0 (Figure 3B) were ∼0.71%, 2.39%, and 3.53% from the original state under shear stress in3–10, 15, 30 dyne/cm^2^ stimulation, respectively. The values kept slowly increasing when the flow stopped. On the contrary, MRC-5 (Figure 3D) shows −0.22%, −0.75%, −2.48%, and −3.33% adhesion changes from initial point under3–7.5, 10, 15, 30 dyne/cm^2^ stimulation, respectively. The values increased to −0.125%, −0.16%, −0.59%, −2.69%, in ∼40 minutes when the flow stopped. The Hs68 cells did not respond significantly to shear stress until 10 dyne/cm^2^ (Figure 3F). About −0.66%, −1.35%, and −2.73% adhesion changes at 10, 15, and 30 dyne/cm^2^ stimulation. There was an small increase in the signal when the flow stopped.

### The Maximum Adhesion Change and Threshold of Shear Stress

**Figure 4:**
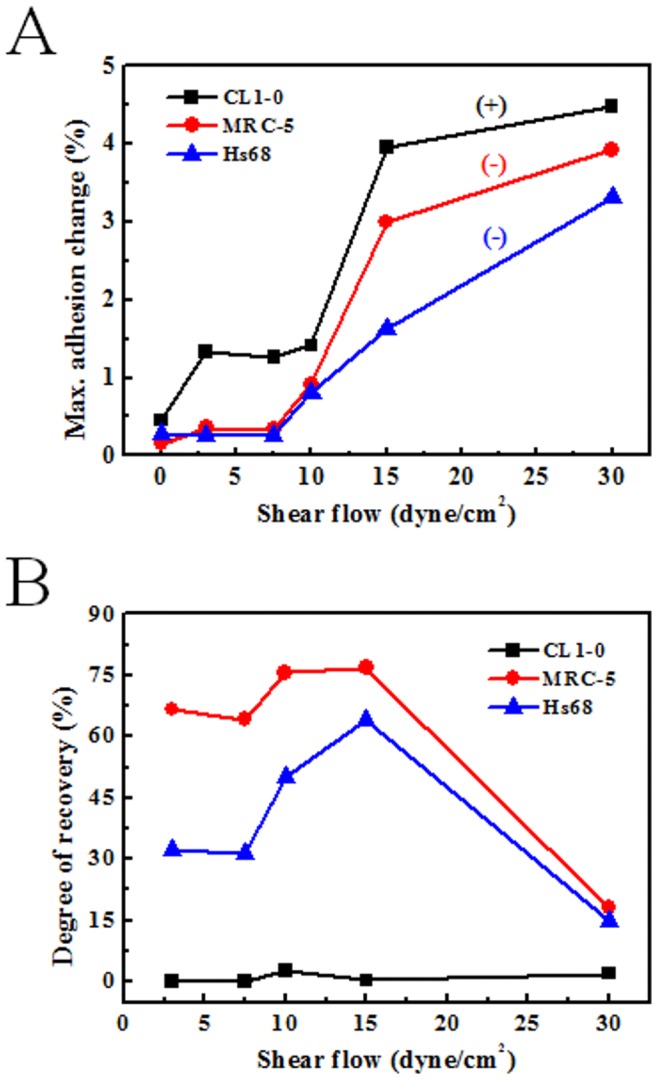
The maximum adhesion changes and the degree of adhesion recovery for different cells under shear flow conditions. “+” indicates increased adhesion, “–”, decreased adhesion.

The maximum adhesion change (*C_max_*) as a function of shear stress for various kinds of cells are shown in Figure 4A. The CL1-0 (cancer cell) has a larger adhesion change than MRC-5 and Hs68 cells. The different response to the shear stress stimulation between cancer cell and normal cell also indicates the malignant phenotype in cancer research. The cancer cell is more rigid than the normal cell which is related to the enhancement of integrin linked focal adhesion complex [43]. In CL1-0 cell, the *C_max_* shows two different stages under shear stress stimulation. There is a significant increase of *C_max_* when the stress is higher than 10 dyne/cm^2^. For MRC-5 and HS68 cells, there are also obvious changes in cell adhesion when shear stress is higher than 10 dyne/cm^2^. Such threshold for the laminar shear stress can be explained by mechanical stress responses in endothelial cells [44]–[46]. When the laminiar shear stess is higher than 5 dyne/cm^2^, cell cytoskeletal and fibronectin are rearranged. When the shear stress reaches 10 dyne/cm^2^, cells are realigned with flow. There is a directional remodeling of focal adhesion sites [45]. The change of cell adhesion results in a large change of SPR signal. Therefore, a threshold near 10 dyne/cm^2^ was measured. In terms of the results presented here, cell adhesion change upon shear force stimulation monitored by nanoslit SPR is in good agreement with the magnitude of shear stress for remodeling of focal adhesions.

### Adhesion Recovery from Shear Stress Stimulation

The strength of recovery from shear stress stimulation is also dependent on types of cells. Figure 4B shows the degree of adhesion recovery (γ) for three different cell lines. The degree of adhesion recovery is defined by
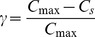
(4)where *C_s_* is the steady state adhesion after stopping the flow. The CL1-0 cancer cell has a very low degree of recovery. It maintains increased adhesion even after stopping the shear flow for 40 minutes. The recovery degree is lower than 5%. On the contrary, the MRC-5 and Hs68 cells have significant recovery degrees. For MRC-5 cells, the recovery degree reaches higher than 70%. It should be noted that the recovery degree substantially decreased when greater shear stress (30 dyne/cm^2^) was applied to the cells. The recovery phenomena was observed both in lung fibroblasts (MRC-5) and skin fibroblasts (Hs68) but was more apparent in MRC-5 cells. We suggest that this is due to the innate tissue function of cells. The lung tissue stretches cyclically for breathing and the cell responds to the shear stress stimulation. It needs a quicker and higher recovery rate when it is mechanically stimulated. On the other hand, the cancer cell gives the adaption to the flowing shear stress, which has been studied in hematological of oncology [47]. The blood circulation system is one of the major routes by which cancer spreads. The shear stress in capillaries of human body is with more than 15 dyne/cm^2^ [48]. The cancer cell travels in the blood vessel, rests in the capillaries region and forms the metastatic tumor (secondary tumor) [49]. The nature of cancer cells, high proliforation rate, may show the advantage to antagonize the mechanical stimulation. They are more adaptive to the shear flow conditions, and thus have a low degree of recovery.

## Conclusions

In summary, we studied the dynamic responses of cells under different laminar shear stresses by using a gold nanoslit array SPR sensor. We successfully measured the response of three types of human cell lines: non-small cell lung cancer (CL1-0), lung fibroblast (MRC-5), and dermal fibroblast (Hs68). Results indicate that cells can sense different strengths of mechanical force in fluidic shear stress. The cancer cell shows an increase of focal adhesion under fluidic shear stress stimulation. On the contrary, the fibroblast cells lose their adhesion in shear flow. The lung fibroblast is more sensitive to shear stress than the skin fibroblast. Both fibroblast cells can be recovered from stimulation. The cell responses to shear stress are different depending on the cell types, which could be linked to the cell function and the mechanism of cytology, pathology, oncology, and tissue engineering. Comparing with other thechiques for mechanobiological study of cells, nanoslit array SPR provides the monitoring both in the response and recovery of cells from shear stress stimulation in label-free and real-time detection. It can be applied to dynamic study of mechanobiology in different physical and chemical microenvironments as well as in the diagnosis of diseases.
